# Distance-dependent aposematism and camouflage in the cinnabar moth caterpillar (*Tyria jacobaeae*, Erebidae)

**DOI:** 10.1098/rsos.171396

**Published:** 2018-02-21

**Authors:** James B. Barnett, Innes C. Cuthill, Nicholas E. Scott-Samuel

**Affiliations:** 1 School of Biological Sciences, University of Bristol, Bristol Life Sciences Building, 24 Tyndall Avenue, Bristol BS8 1TQ, UK; 2 School of Experimental Psychology, University of Bristol, 12a Priory Road, Bristol BS8 1TU, UK

**Keywords:** defensive coloration, warning coloration, background matching, salience, viewing distance, visual acuity

## Abstract

Defended prey often use distinctive, conspicuous, colours to advertise their unprofitability to potential predators (aposematism). These warning signals are frequently made up of salient, high contrast, stripes which have been hypothesized to increase the speed and accuracy of predator avoidance learning. Limitations in predator visual acuity, however, mean that these patterns cannot be resolved when viewed from a distance, and adjacent patches of colour will blend together (pattern blending). We investigated how saliency changes at different viewing distances in the toxic and brightly coloured cinnabar moth caterpillar (*Tyria jacobaeae*). We found that although the caterpillars' orange-and-black stripes are highly salient at close range, when viewed from a distance the colours blend together to match closely those of the background. Cinnabar caterpillars therefore produce a distance-dependent signal combining salient aposematism with targeted background matching camouflage, without necessarily compromising the size or saturation of their aposematic signal.

## 1. Introduction

Aposematic (warning) coloration signals directly to potential predators, warning that attempted predation is likely to be unprofitable [1,2]. Aposematic patterns frequently contain bright colours and high contrast patterning, which have been linked to greater speed and accuracy of predator avoidance learning, and have been hypothesized to increase the saliency of signals across heterogeneous backgrounds [2–4].

Limitations in predator visual acuity, however, mean that when viewed from a distance a pattern can no longer be resolved, and adjacent patches of colour will blend together [5,6]. This distance-dependent pattern blending has the potential to disrupt a predator's ability to recognize an aversive signal [7]. Conversely, though, where the blended colour matches that of the background, pattern blending may instead result in camouflage [5,8,9].

Rather than completely avoiding defended aposematic prey, avian predators manage their intake of nutrients and toxins, and will increase their consumption of defended prey when under nutritional stress or when alternative prey are hard to find [10–14]. The high predator encounter rate associated with high conspicuousness, and the resulting lethal and sub-lethal costs, may favour patterns which maximize neither camouflage nor conspicuousness [15–17].

Low intensity signals, with low colour saturation or small aposematic components, trade off the costs and benefits of camouflage and salient aposematism [15–17]. Distance-dependent signalling offers an alternative, which maintains colour saturation and allows control of the distances at which camouflage and aposematism function [7–9,16,18–20].

**Figure 1.**
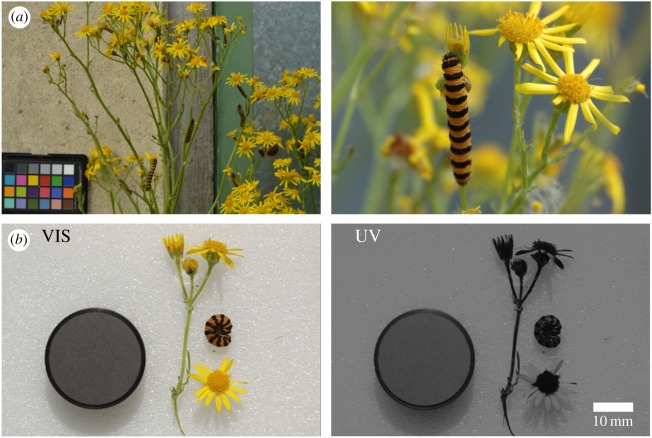
The cinnabar moth caterpillar and its ragwort food plant. (*a*) The cinnabar caterpillar *in situ* (left—image used for visual modelling, right—close up of feeding behaviour). (*b*) Caterpillar and ragwort photographed under human visible (left) and ultraviolet (right) light, with a 15% reflectance Spectralon® grey standard. There is minimal UV reflectance from the caterpillar and the ragwort stem, but there is high UV reflectance from the ragwort petals (appearing white in the UV image).

It has been suggested that distance-dependent effects can be mediated by the addition of small aposematic spots to an otherwise cryptic pattern [19,20], by combining high spatial frequency aposematic patterns with low spatial frequency camouflage [16], or by pattern blending, where adjacent colour patches blend together to form a cryptic mean colour [7–9]. Here, we add ecological validity to the pattern blending hypothesis by investigating whether the striped aposematic pattern of the cinnabar moth caterpillar (*Tyria jacobaeae*, Erebidae) could constitute camouflage when viewed from a distance (figure 1*a*).

Cinnabar caterpillars sequester toxic alkaloid compounds from a specialized diet of ragwort (*Jacobaea* spp., Asteraceae), and advertise their defence with highly contrasting orange-and-black stripes (figure 1) [21]. We used computational visual modelling of a UV-sensitive passerine bird to investigate how the perception of caterpillar coloration changes at different distances.

## 2. Material and methods

### 2.1. Photography

In August 2014, wild final-instar cinnabar caterpillars were photographed in suburban green space within the city of Bristol, UK. Caterpillars and their natural food plant were photographed under natural, daylight, illumination with a UV sensitive Nikon D70 Digital SLR camera and UV-NIKKOR 105 mm lens (Nikon Corporation, Tokyo, Japan), with appropriate VIS filters, and a 15% reflectance Spectralon® grey standard (Labsphere, Inc. North Sutton, NH, USA).

UV photography revealed minimal UV reflectance from the caterpillar and the ragwort stem, although there was high UV reflectance from the ragwort flowers' outer petals (figure 1*b*). This negligible UV reflectance from the stem allowed the tetrachromatic vision of a passerine bird (see below) to be modelled from standard, but calibrated, RGB photography. As the caterpillars are not usually found on the UV reflecting petals, these were excluded from the analysis.

Caterpillars (*n* = 10) were photographed from a distance of approximately 30 cm, *in situ*, in their natural feeding position on ragwort stems, with a Nikon D3200 Digital SLR camera and AF-S DX NIKKOR 35 mm prime lens (Nikon Corporation, Tokyo, Japan). All images contained a ColorChecker Passport (X-Rite Inc. Grand Rapids, MI, USA) for calibration (figure 1*a*—left).

### 2.2. Image analysis

Raw (NEF) images were converted to 8-bit TIFF files at 300 dpi. These images were calibrated, linearized [22], and size-scaled using the ColorChecker Passport, and the coordinates corresponding to the caterpillar and the ragwort stem were labelled by hand in MATLAB 2015a (The Mathworks Inc. Natick, MA, USA). Up to 1000 pixels from the background and 1000 pixels from the caterpillar were then selected, without replacement, using MATLAB's random number generator, for analysis (sometimes there were slightly fewer than 1000 pixels in a caterpillar).

Visual modelling used cone sensitivity, oil droplet and ocular media data from the UV-sensitive, tetrachromatic European starling (*Sturnus vulgaris*, Sturnidae), with single cone peak absorption (*λ*_max_) of 563 nm (LWS), 504 nm (MWS), 449 nm (SWS), 362 nm (UVS), and as a surrogate for luminance, double cones (D) with a *λ*_max_ of 563 nm [23].

Opponent processing is central to colour perception in humans [24], and the L*a*b* colour space is internationally recognized as a standard for human colour perception (CIELAB, 1979). L*a*b* was developed by psychophysical testing, and attempts to match the perceived difference between two colours to the linear separation of their locations in the colour space. Colour is split into a measure of luminance or achromatic intensity (L), and two orthogonal dimensions that describe hue. These latter opponent channels, red-green (a*) and yellow-blue (b*), are consistent with both perception and what we know of neural processing [25,26]. Equivalent psychophysical testing has been far less intensive for birds, but there is evidence of a comparable system of opponent channels [27–29]. As UV information was negligible, we assessed colour in a three-dimensional colour space, following the logic of L*a*b*, made up of luminance, red-green and yellow-blue [17,30].

Luminance was measured from the stimulation of the D cone; red-green opponency was produced from the relative stimulation of the LWS and MWS cones, and yellow-blue opponency was produced from the relative stimulation of the combined LWS and MWS cones to the SWS cone. Indeed, other colour channels could be modelled, and we make no claim that birds have these colour channels, but as they are orthogonal dimensions they are efficient descriptors of the colour space [17,30].

The effect of viewing distance was represented using a 2D Gaussian filter for pattern blending (function *imgaussfilt* in MATLAB 2015a). Two distances were modelled: close-range (High: the pixel resolution of the photographs) and from beyond the resolution limit of the pattern (Low: a Gaussian filter with a standard deviation of half a caterpillar length). As contrast sensitivity and visual acuity are dependent on absolute light levels, which vary continuously under field conditions, we do not specify any particular viewing distance. Rather, these two conditions ensure that we can represent colour perception within and beyond the resolution limit of the pattern, without obscuring the whole caterpillar. Data are available in Dryad [31].

Colour discrimination was assessed using binomial general linear mixed models, fitted using function *glmer* in the R package *lme4* [32]. We used leave-one-out-cross-validation to avoid overfitting, testing each caterpillar in turn against a model developed (‘trained’) using the other nine. Sensitivity (proportion of correct caterpillar classifications) was then extracted using the R package *caret* [33].

## 3. Results

**Figure 2.**
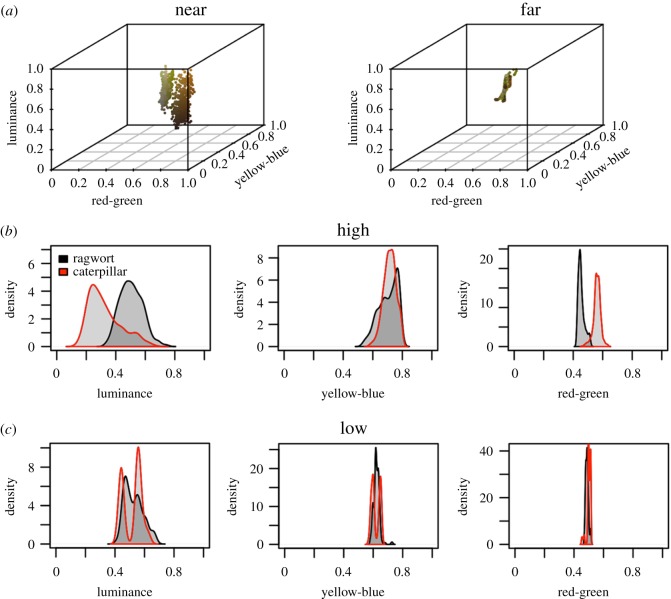
The cinnabar moth caterpillar (*n* = 10) and the ragwort stem (*n* = 10) as viewed by a model of avian colour vision. (*a*) The caterpillar and ragwort at high (left) and low (right) resolution showing the pixel colours in three-dimensional avian colour space. Individual channel responses at high (*b*) and low (*c*) spatial resolution. At full resolution, the caterpillar and its food plant are easily distinguished, however at low spatial resolution the distinction between caterpillar and background is greatly reduced.

We found that the distinction between the caterpillar and the ragwort stem changed at different spatial resolutions. At high spatial resolutions, equivalent to close-range viewing, the caterpillar pixels were easily distinguished from the background across luminance and the red-green opponent channel (figure 2*a* left, *b*). At lower spatial resolutions, corresponding to viewing the caterpillar from distances beyond the resolution limit of the pattern, the distinction between caterpillar and background decreased across all three channels (figure 2*a* right, *c*). The proportion of caterpillar colours misclassified as belonging to ragwort (based on cross-validated predictions from GLMMs) increased from 1% at high resolution, to 13% at low resolution, to 30% for the average colours.

## 4. Discussion

It has been hypothesized that the orange-and-black stripes of the cinnabar caterpillar may provide both aposematism and camouflage [34]. We found strong UV reflectance from the petals of the ragwort flower, but negligible UV reflectance from the caterpillars. Thus, although the orange-yellow colours of the cinnabar stripes might, to a human, look superficially like the flower colours, they do not match in the eyes of UV sensitive predators such as many birds [23,35] and hymenopterans [36]. In fact, it is perhaps unsurprising that the caterpillar is not camouflaged via background matching to the petals because the caterpillars are usually found on the stems. Note, however, that we cannot rule out other camouflage effects, such as disruptive coloration, or the possibility of matching components of the wider visual scene. That said, our results hold true for the most relevant background, the stems on which the caterpillars usually reside (J. B. Barnett 2014, personal observation).

Avian visual modelling showed that at close range there is a large difference between the colour of the caterpillar and its background. At greater distances, however, the orange-and-black components are blended, greatly reducing this distinction. As distance increases it becomes more difficult to distinguish a target from a background, due to the decreasing size of the target's image on the retina and the increasing summation of adjacent elements within the visual scene. These data suggest that the pattern of the cinnabar caterpillar accelerates this process by summating to a cryptic colour: this effect is produced at distances where the caterpillar itself is still resolvable. In a previous experiment using artificial caterpillars, we identified that this process of pattern blending can reduce detectability at greater distances, while retaining an effective aposematic defence, leading to an increase in survival over more conspicuous and more cryptic patterns [9].

It has also been suggested that the caterpillars of the swallowtail (*Papilio machaon*, Papilionidae) and apollo (*Parnassius apollo*, Papilionidae) butterflies produce distance-dependent patterns, which are neither maximized for camouflage nor maximised for conspicuousness [19,20]. These studies found that the small aposematic spots present on the otherwise camouflaged pattern are only visible at close range. However, the small size of the spots may limit their aversive properties [2]. In contrast, the cinnabar caterpillar produces a large and highly saturated aposematic pattern at close range which would be expected to offer a more salient aversive signal [2,4].

It is relevant, when considering the optimal pattern for signalling (or being inconspicuous) at different distances, that contrast sensitivity is greater for luminance than colour at high spatial frequencies [37,38]. That is, a bird will detect contrast in luminance further away, or in smaller objects/patterns, than contrast in hue. The cinnabar caterpillar has patterns with high modulation in both hue and luminance, but other species in other ecological contexts may use colour patterns more subtly and strategically to vary the distance-dependent information.

It is well known that many caterpillars are camouflaged, or have less saturated colours, during their early instars, and switch to aposematic coloration once their chemical defences have developed [39–41]. The spatial frequency of the cinnabar caterpillars' striped pattern decreases (stripes get thicker) as the caterpillar grows, and consequently the distance at which the pattern can be resolved increases. This may allow for a shifting balance between camouflage and aposematism as the caterpillar increases in size and sequesters greater concentrations of toxins. Moreover, retaining the same pattern may allow early instar caterpillars to gain protection from mimicking the coloration of later stage caterpillars which, presumably, carry a greater toxin load.

High contrast striped patterns have repeatedly been demonstrated to increase the saliency of aposematic signals [2–4]. We provide evidence that, in the case of the cinnabar caterpillar, high contrast stripes may also reduce the long-range detectability of the aposematic signal. These data are consistent with striped patterns decreasing long-range detectability and increasing survival, as previously shown [9], but do not of course rule out other functions (e.g. memorability or discriminability from palatable prey). There is, however, no reason to assume that all high contrast patterns will blend to match the colour of the background, and therefore our study could have rejected (or at least rendered untenable) the hypothesis of distance-dependent camouflage. Further data are needed to isolate the multiple ways in which the patterning affects *in situ* predation rates [7,9]. As striped patterns are a widespread component of aposematic signals, this camouflaging effect may be underappreciated in studies of signal design and may apply more widely across seemingly conspicuously coloured taxa.

## Data accessibility

Raw data can be accessed from the Dryad Digital Repository (http://dx.doi.org/10.5061/dryad.2rc5d) [31].
